# Does antenatal cholecalciferol supplementation affect the mode or timing of delivery? Post hoc analyses of the MAVIDOS randomized controlled trial

**DOI:** 10.1093/pubmed/fdac160

**Published:** 2022-12-28

**Authors:** Rebecca J Moon, Stefania D’Angelo, Sarah R Crozier, Elizabeth M Curtis, Michelle Fernandes, Alexandra J Kermack, Justin H Davies, Keith M Godfrey, Nicholas J Bishop, Stephen H Kennedy, Ann Prentice, Inez Schoenmakers, Robert Fraser, Saurabh V Gandhi, Hazel M Inskip, Muhammad Kassim Javaid, Aris T Papageorghiou, Cyrus Cooper, Nicholas C Harvey

**Affiliations:** MRC Lifecourse Epidemiology Centre, University of Southampton, Southampton, UK; Paediatric Endocrinology, University Hospital Southampton National Health Service (NHS) Foundation Trust, Southampton, UK; MRC Lifecourse Epidemiology Centre, University of Southampton, Southampton, UK; MRC Lifecourse Epidemiology Centre, University of Southampton, Southampton, UK; MRC Lifecourse Epidemiology Centre, University of Southampton, Southampton, UK; MRC Lifecourse Epidemiology Centre, University of Southampton, Southampton, UK; Department of Women’s Health, University Hospital Southampton NHS Foundation Trust, Southampton, UK; Paediatric Endocrinology, University Hospital Southampton National Health Service (NHS) Foundation Trust, Southampton, UK; MRC Lifecourse Epidemiology Centre, University of Southampton, Southampton, UK; NIHR Southampton Nutrition Biomedical Research Centre, University of Southampton and University Hospital Southampton NHS Foundation Trust, Southampton, UK; Academic Unit of Child Health, Sheffield Children’s Hospital, University of Sheffield, Sheffield, UK; Nuffield Department of Women’s & Reproductive Health, John Radcliffe Hospital, University of Oxford, Oxford, UK; MRC Epidemiology Unit, University of Cambridge, previously at MRC Human Nutrition Research, Elsie Widdowson Laboratory, Cambridge, UK; Faculty of Medicine and Health Sciences, Department of Medicine, University of East Anglia, Norwich, UK; Department of Obstetrics and Gynaecology, Sheffield Hospitals NHS Trust, University of Sheffield, Sheffield, UK; Department of Obstetrics and Gynaecology, Sheffield Hospitals NHS Trust, University of Sheffield, Sheffield, UK; MRC Lifecourse Epidemiology Centre, University of Southampton, Southampton, UK; NIHR Southampton Nutrition Biomedical Research Centre, University of Southampton and University Hospital Southampton NHS Foundation Trust, Southampton, UK; National Institute for Health Research (NIHR) Musculoskeletal Biomedical Research Centre, University of Oxford, Oxford, UK; Nuffield Department of Women’s & Reproductive Health, John Radcliffe Hospital, University of Oxford, Oxford, UK; MRC Lifecourse Epidemiology Centre, University of Southampton, Southampton, UK; NIHR Southampton Nutrition Biomedical Research Centre, University of Southampton and University Hospital Southampton NHS Foundation Trust, Southampton, UK; National Institute for Health Research (NIHR) Musculoskeletal Biomedical Research Centre, University of Oxford, Oxford, UK; MRC Lifecourse Epidemiology Centre, University of Southampton, Southampton, UK; NIHR Southampton Nutrition Biomedical Research Centre, University of Southampton and University Hospital Southampton NHS Foundation Trust, Southampton, UK

**Keywords:** 25-hydroxyvitamin D, Caesarean, cholecalciferol, delivery, labour, post-partum haemorrhage, pregnancy, preterm birth

## Abstract

**Background:**

Observational studies relating maternal 25-hydroxyvitamin D status to timing and mode of delivery have reported inconsistent results. We assessed the effect of antenatal cholecalciferol supplementation on the incidence of preterm birth, delivery mode and post-partum haemorrhage (PPH).

**Methods:**

MAVIDOS was a randomized, double-blind, placebo-controlled trial of 1000 IU/day cholecalciferol from 14 weeks’ gestation until delivery. Gestational age, mode of delivery [categorized as spontaneous vaginal delivery (SVD), instrumental (including forceps and vacuum extraction) or Caesarean section] and PPH (>500 ml estimated blood loss) were determined from medical records.

**Results:**

A total of 965 women participated in the study until delivery. Gestation at birth and incidence of preterm birth (cholecalciferol 5.7%, placebo 4.5%, *P* = 0.43) were similar between the two treatment groups. SVD (versus instrumental or Caesarean delivery) was more likely in women randomized to cholecalciferol [Relative Risk (RR) 1.13, 95% confidence interval (CI) 1.02,1.25] due to lower instrumental (RR 0.68, 95%CI 0.51,0.91) but similar risk of Caesarean delivery (RR 0.94, 95%CI 0.74,1.19). PPH was less common in women randomized to cholecalciferol [32.1% compared with placebo (38.1%, *P* = 0.054) overall], but similar when stratified by delivery mode.

**Conclusions:**

Antenatal cholecalciferol supplementation did not alter timing of birth or prevalence of preterm birth but demonstrated a possible effect on the likelihood of SVD.

## Background

Vitamin D deficiency in pregnancy is common. In a study of predominately White women in the south of the UK, 31% had a serum 25(OH)D < 50 nmol/l (typically considered ‘insufficient’^1^) and 18% < 25 nmol/l (typically considered ‘deficient’^1^) in late pregnancy.^2^ In a more ethnically diverse population in London, 36% women had 25(OH)D < 25 nmol/l in early pregnancy.^3^ Reports of similarly high prevalence of vitamin D deficiency in pregnancy have also been reported in other countries across Europe,^4–6^ although in Nordic countries where ultraviolet B (UVB) exposure is limited, the reported prevalence of vitamin D deficiency is lower than in some Southern European countries. This reflects higher supplement use and food fortification practices, highlighting the importance of dietary intake to maintain vitamin D status.^7^

The primary function of vitamin D is in calcium and phosphate homeostasis and severe maternal vitamin D deficiency can result in neonatal hypocalcaemia resulting in seizures, rickets and cardiomyopathy. There is consistent evidence that the incidence of symptomatic neonatal hypocalcaemia can be reduced by antenatal vitamin D supplementation.^8–10^ In the UK, all pregnant women are advised to take 400 IU/day vitamin D throughout pregnancy.^11^ Similar guidelines also exist in other developed countries.^12–14^ It has also been proposed that 25(OH)D might have other pleiotropic functions. Indeed, the vitamin D receptor is expressed in a wide range of tissues and local conversion of 25(OH)D into the active metabolite 1,25(OH)_2_D occurs with auto and paracrine effects, including in the myometrium^15^ and placenta.^16^^,^^17^

Vitamin D deficiency has been associated with obstetric outcomes in numerous observational studies, including timing and mode of delivery and incidence of post-partum haemorrhage (PPH),^18^^,^^19^ but the study findings are inconsistent.^20^^,^^21^ For example, maternal vitamin D deficiency has been associated with an increased risk,^22–24^ no difference in risk^25–27^ and reduced risk of preterm birth.^28^^,^^29^ Furthermore, a recent meta-analysis of observational studies suggested that the timing of vitamin D deficiency may be important to the risk of preterm birth, with only deficiency in the second, and not the third, trimester being of potential importance to this outcome.^30^ Several recent observational studies have also shown lower maternal 25(OH)D levels in those requiring Caesarean section compared with vaginal delivery,^31–34^ but observational studies can be confounded by factors that affect both maternal 25(OH)D and risk of needing an operative delivery, such as maternal obesity, gestational weight gain and ethnicity.^35^ Christoph *et al.* found vitamin D deficiency reduced the incidence of PPH in an observational study.^19^ In contrast, women in China with gestational diabetes (GDM) who received vitamin D supplementation had a reduced risk of PPH.^36^ Importantly, observational studies have variably adjusted for recognized risk factors for these outcomes. For example, risk factors for preterm delivery include amongst others grand multiparity, previous preterm birth, low socioeconomic status, GDM, hypertension, vaginal infections, smoking and alcohol use.^37^ Risk factors for instrumental delivery include nulliparity, use of epidural analgesia, older maternal age^38^^,^^39^ and for PPH are related to increased risk of poor uterine contraction (e.g. polyhdramnios, multiple birth, rapid labour, infection), retained products of conception, trauma or coagulopathy.^40^

Despite the wealth of observational evidence, there are few data from intervention studies to support the use of vitamin D supplementation to reduce the incidence of preterm birth and rates of Caesarean section.^41^ Furthermore, given the higher risk of PPH in operative and instrumental deliveries,^42^ it is unknown if PPH risk can be modified by pregnancy vitamin D supplementation, either directly or indirectly due to the effect on delivery mode. We assessed, in this post hoc analysis, the effect of antenatal cholecalciferol supplementation on the timing and mode of delivery and incidence of PPH in a randomized, placebo-controlled trial.^43^

## Methods

### The maternal vitamin D osteoporosis study

The Maternal Vitamin D Osteoporosis Study (MAVIDOS) was a multicentre, double-blind, randomized, placebo-controlled trial of vitamin D supplementation in pregnancy. The primary outcome was neonatal bone mass. A detailed description of the study methods^43^ and primary findings have been published previously.^44^

Women attending one of three United Kingdom (UK) hospitals [University Hospital Southampton National Health Service (NHS) Foundation Trust, Southampton, UK (latitude 50.9° North); Oxford University Hospitals NHS Foundation Trust, Oxford, UK (latitude 51.8° North); Sheffield Hospitals NHS Trust (University of Sheffield), Sheffield, UK (latitude 53.4° North)] for early pregnancy ultrasound screening (11–14 weeks’ gestation) between 6 October 2008 and 11 February 2014 were invited to participate in the study. Gestational age was determined using the date of last menstrual period (LMP) and with first trimester foetal ultrasonographic crown-rump length measurement used if >7 days’ discrepancy between LMP and scan dates, uncertain LMP date, irregular cycles or hormonal contraception-use within last 3 months. Inclusion criteria were age over 18 years, singleton pregnancy and gestational age ˂17 weeks based on LMP and ultrasound measurements. Women with known metabolic bone disease, renal stones, hyperparathyroidism or hypercalciuria, those taking medication known to interfere with foetal growth, foetal anomalies on ultrasonography and women already using >400 IU/day vitamin D supplementation were excluded. A screening blood sample was obtained and analysed on the local NHS platform [all three laboratories (Southampton, Oxford and Sheffield) participate in the Vitamin D External Quality Assessment Scheme (DEQAS) vitamin D quality assurance system (http://www.deqas.org/)]. Women with 25(OH)D between 25 and 100 nmol/l and serum calcium <2.75 mmol/l were eligible to enrol fully in the study.

### Intervention

Participants were randomized to either cholecalciferol 1000 IU/day or matched placebo [Merck KGaA, (Darmstadt, Germany)/Sharp Clinical Services (Crickhowell, UK; previously DHP-Bilcare)], commenced before 17 weeks’ gestation. Packs of medication were randomly assigned in a 1:1 ratio by a computer-generated sequence in randomly permuted blocks of 10, starting randomly midway through the block, and sequentially numbered, before delivery to the study sites, and then dispensed in order by each study pharmacist. The study medication was provided in a single box containing all medication for the whole pregnancy. The participants, individuals providing antenatal and intrapartum care, and all field researchers involved in data collection and sample analysis were blinded to the assignment of the intervention. All participants received standard antenatal care, and could continue self-administration of dietary supplements containing up to 400 IU/day vitamin D. Women wishing to take dietary supplements containing >400 IU/day vitamin D were excluded from participation in the study, and those who increased their personal supplementation use above this threshold during the study were excluded from the analysis.

### Outcomes

#### Maternal assessments during pregnancy

Prior to commencing the study medication, and again at 34 weeks’ gestation, the women attended the research centre for a detailed assessment lifestyle and health (smoking, medical history, current medication use) and use of vitamin D supplementation using interviewer-led questionnaires. Height and weight were measured and used to calculate body mass index (BMI). Compliance with study medication was assessed by pills counts.

#### Assessment of 25(OH)D

Non-fasted venous blood samples were obtained on the day that the study medication was dispensed and at 34 weeks’ gestation. Serum was stored at −80°C. 25(OH)D concentration was assessed by chemiluminescence immunoassay (Liaison automated platform, Diasorin, Minnesota, USA). All samples were analysed in a single batch at the end of the study at Medical Research Council (MRC) Human Nutrition Research, Cambridge, UK. Within- and between-assay coefficients of variation were 4.1 and 6.1%. Details of assay performance and quality control through participation in DEQAS and calibration against the National Institute of Standards and Technology (NIST) standards are given elsewhere.^45^^,^^46^

#### Delivery and infant details

Gestational age at birth and mode of delivery were collected by a research nurse/midwife from participants’ medical records. Preterm birth was defined as delivery before 37 weeks’ completed gestation. Mode of delivery was categorized as spontaneous vaginal delivery (SVD), instrumental vaginal delivery (i.e. forceps and/or vacuum extraction) or Caesarean section (emergency or elective). When a woman started labour spontaneously before the date of a planned Caesarean section and still delivered by Caesarean section, this was categorized as an elective Caesarean. Since delivery mode is associated with differences in blood loss, estimated blood loss (EBL) was extracted from the medical records; PPH was defined as an EBL ≥ 500 ml, with major PPH as ≥1000 ml.^42^ The obstetric team were not involved in this research study and were also blinded to the allocation to cholecalciferol or placebo. Sex and birth weight were also extracted from the medical records.

### Statistical analysis

The analysis performed here was post hoc exploratory analysis that was not stated in the original trial protocol.^43^ The analysis was performed on an intention-to-treat basis. Comparisons between treatment groups were performed using *t*-tests for normally distributed continuous outcomes, Mann–Whitney *U*-tests for non-normally distributed data and χ^2^ tests for categorical variables. Poisson regression with robust standard errors was used to calculate the relative risk of each delivery mode in comparison to all alternative delivery modes; as such Caesarean section was compared with women who did not have a Caesarean section (SVD or instrumental combined) and SVD was compared with those not achieving an SVD (Caesarean section and instrumental combined). This statistical approach will correct estimates in the case of binary outcomes,^47^ but the estimates and confidence intervals were the same when repeated using log-binominal regression models. Given the study design and balanced characteristics of the mothers at baseline, results are presented unadjusted for any covariates. In exploratory analyses, we included adjustment for compliance with study medication and assessed for interactions between treatment allocation and maternal age or baseline 25(OH)D status at randomization. All analyses were performed using Stata v14.2 (Statacorp, College Station, TX, USA).

**Fig. 1:**
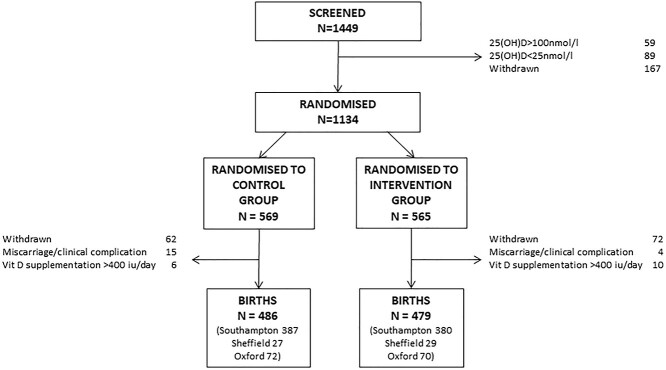
Consort diagram.

### Ethics approval

The study was approved by the Southampton and South-West Hampshire Research Ethics Committee. MAVIDOS was registered prospectively (ISRCTN:82927713; EUDRACT:2007-001716-23); full approval from UK MHRA was granted, and written, informed consent was obtained from all participants.

## Results

A total of 1449 women consented to baseline 25(OH)D screening to determine the eligibility to participate in the full trial; 59 and 89 women were excluded due to 25(OH)D < 25 nmol/l and >100 nmol/l, respectively. A further 167 women withdrew prior to randomization. A total of 1134 women were initially randomized, and 965 continued in the study until delivery (Fig. 1) with similar proportions in each treatment group at each study centre. Maternal characteristics are shown in Table 1. 25(OH)D was similar at baseline in the two groups, but higher in women randomized to cholecalciferol [68.2 nmol/l (standard deviation {SD} 21.9 nmol/l)] than placebo [43.4 nmol/l (SD 22.4 nmol/l)] at 34 weeks’ gestation (*P* < 0.001). Compliance with study medication was high in both groups (placebo: median 95.0%, Interquartile range (IQR) 88.2,98.8%; cholecalciferol: median 96.2% IQR 88.9,99.2%). Maternal weight gain from early to late pregnancy did not differ between the two groups [placebo: mean 9.45 kg (SD 3.65 kg); cholecalciferol: mean 9.57 kg (SD 3.55 kg), *P* = 0.63].

**Table 1:** Characteristics of the women

	Placebo	Cholecalciferol
N	486	479
Age (years), mean (SD)	30.7 (5.3)	30.8 (5.1)
Smoking at randomization, %	7.9	8.2
Nulliparous, %	43.8	42.1
BMI at randomization (kg/m^2^), median (IQR)	25.6 (22.9–29.9)	24.6 (22.3–28.6)
Height (cm), mean (SD)	165.7 (6.6)	165.4 (6.3)
White ethnicity, %	94.6	95.2
25(OH)D at randomization (nmol/l), mean (SD)	45.7 (16.9)	46.8 (17.4)
Participation in moderate/strenuous physical activity in late pregnancy, N (%)	280 (67.8)	267 (67.9)
Use of additional vitamin D supplementation (up to 400 IU/day) during pregnancy, N (%)	119 (27.5)	120 (29.1)
Season of delivery, N (%)		
Winter (December–February)	102 (21.0)	104 (21.7)
Spring (March–May)	126 (25.9)	120 (25.1)
Summer (June–August)	130 (26.8)	122 (25.5)
Autumn (September–November)	128 (26.3)	133 (27.8)

### Gestational age and birth weight at delivery

The proportion of male infants born in each group was similar (placebo 51.7%, cholecalciferol 53.9%, *P* = 0.49). Median gestational age at delivery was 40.3 weeks (IQR 39.3, 41.1 weeks) in women randomized to placebo and 40.3 weeks (IQR 39.1, 41.0 weeks) in those randomized to cholecalciferol (*P* = 0.22). The incidence of preterm birth was also similar (placebo 4.5%, cholecalciferol 5.7%, *P* = 0.43). Birthweight did not differ between the two groups [placebo: mean 3518 g (SD 517 g); cholecalciferol: 3481 g (SD 543 g), *P* = 0.28]. Occipitofrontal circumference also did not differ [placebo: mean 35.5 cm (SD 1.5 cm); cholecalciferol: 35.4 cm (SD 1.4 cm), *P* = 0.62].

### Mode of delivery

**Fig. 2:**
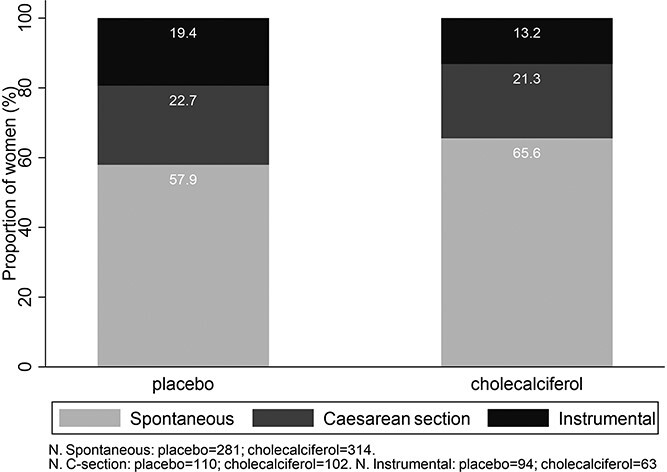
Mode of delivery in women randomized to placebo or 1000 IU/day cholecalciferol during pregnancy (*P* = 0.03).

Mode of delivery differed between the two groups (*P* = 0.016, Fig. 2); SVD was achieved in 65.6% of women in the cholecalciferol group compared with 57.9% in the placebo group [Relative Risk (RR) 1.13, 95% confidence interval (CI) 1.02,1.25]. The difference results from fewer instrumental deliveries in the cholecalciferol group (13.2%) compared with placebo group (19.4%, RR 0.68, 95%CI 0.51,0.91), whereas delivery by Caesarean section was similar in the two groups (cholecalciferol 21.3%, placebo 22.7%). The overall risk of Caesarean section as opposed to a vaginal (spontaneous or instrumental delivery) was not reduced by cholecalciferol supplementation (RR 0.94, 95%CI 0.74,1.19). Sixty-seven women had an elective section and for 21 women the type of Caesarean section was not documented. The findings were similar when these women were excluded, and inclusion of research centre in the models did not alter the findings. The findings were also similar when compliance with the study medication was included in the models. In exploratory analysis, there was no statistical interaction between treatment allocation and maternal age or baseline 25(OH)D status.

### Post-partum haemorrhage

PPH occurred in 32.1% of women randomized to cholecalciferol and 38.1% of women randomized to placebo (RR 0.84, 95%CI 0.71,1.00). Findings were similar for major PPH, with wider confidence limits for this less frequent outcome (RR 0.77, 95%CI 0.52,1.15). Overall, PPH was more common in women requiring a Caesarean section (60.0%) or instrumental delivery (55.4%) compared with those who had a SVD (21.0%) (*P* < 0.001) but there was no evidence of a statistical interaction between treatment group and delivery mode on risk of PPH (Table 2).

**Table 2:** Proportion of women experiencing post-partum haemorrhage (PPH) by randomization group, stratified by delivery mode

		**Placebo**		**Cholecalciferol**		**Relative risk**
		Total *n*	PPH, *n* (%)	Total *n*	PPH, *n* (%)	
**All**		483	184 (38.1)	476	153 (32.1)	0.84 (0.71, 1.00)
**SVD**		278	62 (22.3)	313	62 (19.8)	0.89 (0.65, 1.21)
**Instrumental vaginal delivery**		94	51 (54.3)	63	36 (57.1)	1.06 (0.79, 1.40)
**Caesarean section**		110	71 (64.6)	100	55 (55.0)	0.85 (0.68, 1.07)
	***Emergency Caesarean section***	63	39 (61.9)	59	32 (54.2)	0.88 (0.65, 1.19)

Information on mode of delivery for one woman was missing.

## Discussion

### Main findings of this study

In this randomized, placebo-controlled trial, 1000 IU/day cholecalciferol during pregnancy in women with an early pregnancy 25(OH)D of 25–100 nmol/l did not alter the gestational age at delivery or the incidence of preterm birth. However, our findings suggest that antenatal vitamin D supplementation might be effective at reducing the need for an instrumental delivery and as a result the associated risk of PPH.

### What is already known on this topic

A systematic review of intervention studies of vitamin D supplementation has not shown that supplementation reduces the risk of preterm birth.^48^^,^^49^ This is in contrast to many observational studies^30^ where findings may be affected by confounding and reverse causality. For example, hospital admission and reduced physical activity in women with threatened preterm birth may result in low serum 25(OH)D due to reduced environmental sunlight exposure. Yonetani *et al*. demonstrated that in women requiring hospitalization for at least 28 days for threatened preterm labour during the second trimester, 25(OH)D reduced from the second to the third trimester by a mean of 13 nmol/l compared with no change in 25(OH)D over the same time period in pregnant women not requiring admission matched for age and season.^50^

### What this study adds

Supplementation with 1000 IU/day cholecalciferol did result in a difference in mode of delivery. The proportion of women having a SVD in those randomized to cholecalciferol was higher than the placebo group with fewer instrumental deliveries but no difference in Caesarean section. As instrumental delivery is associated with increased risk of perineal trauma, maternal psychological distress and infant morbidity (for example trauma, jaundice, facial nerve injury, intracranial haemorrhage), vitamin D supplementation might reduce these outcomes, although we were not able to assess this directly. Corcoy *et al.* also did not find a reduced rate of Caesarean section following supplementation with 1600 IU/day cholecalciferol compared or placebo.^51^ Yap *et al.* found no difference in delivery mode in women with an increased risk of GDM randomized to 5000 IU/day cholecalciferol compared with 400 IU/day.^52^ Hollis *et al.* found that the proportion of women that achieved a SVD was greater in those randomized to 2000 IU/day or 4000 IU/day during pregnancy compared with 400 IU/day.^53^ Variation in findings likely reflects differences in study design including the populations studied, timing of commencement of supplementation and study size/power.

The mechanism by which vitamin D might increase SVD rates could result from effects on uterine contractility and muscle strength. The vitamin D receptor (VDR) has been isolated in human myometrium,^15^ placenta^17^ and skeletal muscle^54^ and vitamin D supplementation results in a small increase in skeletal muscle strength in non-pregnant adults.^55^ Although the role of the VDR in smooth muscle is less certain, calcium status is important to contractility and thus may represent an indirect action of vitamin D on myometrium function. Through this effect on contractility, vitamin D deficiency might reduce abdominal wall and/or pelvic muscle floor strength. In one observational study, women with vitamin D deficiency in late pregnancy had lower pelvic floor muscle strength at 8–10 weeks post-partum independent of delivery mode.^56^ Stafne *et al.* showed that vitamin D deficiency during pregnancy was associated with higher rates of urinary incontinence in mid-pregnancy.^57^ However, in these observational studies, findings could be confounded by overall health and physical activity influencing muscle strength, pelvic floor function and vitamin D status. Low pelvic muscle strength has been associated with prolonged first stage of labour in an observational study of 93 women undergoing induction of labour,^58^ and in a randomized controlled trial pelvic training reduced the frequency of prolonged second stage of labour.^59^ Prolonged labour may increase the need for operative intervention although the exact association of pelvic muscle strength with need for operative delivery remains uncertain.^58^^,^^59^ Two studies have previously examined the relationships between maternal 25(OH)D status and prolonged labour. Gernand *et al.* did not identify an increased risk of prolonged first or second stage in women with a low 25(OH)D concentration measured before 26 weeks’ gestation,^60^ whereas Scholl *et al*. found an ⁓2-fold greater risk of prolonged labour in women with 25(OH)D < 30 nmol/l in early pregnancy compared with those with a 25(OH)D level 50-125 nmol/l.^61^

The observed reduction in PPH by cholecalciferol likely reflects the difference in delivery mode. PPH risk is higher in instrumental and operative deliveries,^42^ and the proportion of women experiencing a PPH in each randomization group was similar when stratified by delivery mode, although statistical power would be reduced to demonstrate this. Recent meta-analysis of two studies of vitamin D supplementation in women with GDM has also suggested pregnancy vitamin D supplementation might reduce PPH.^36^ Furthermore, in the NiPPeR randomized placebo-controlled trial of periconception and pregnancy myo-Inositol, probiotics and micronutrient (including vitamin D) supplementation, major PPH was also reduced by the trial product, despite similar number of women requiring Caesarean section.^62^ Although it is difficult to know which nutritional element(s) contributed to the reduction in PPH in NiPPeR, taken together these findings highlight the need for assessment of PPH in other trials of vitamin D supplementation.

As these are hypothesis-generating post hoc analyses, and the reasons for operative delivery were not documented, future trials would need to focus on delivery characteristics, such as labour timings, use and reasons for any intervention and analgesia, both to confirm our findings and attempt to elucidate the underlying mechanisms. Future studies should additionally aim to establish the benefits of vitamin D supplementation on these outcomes in specific risk groups for both vitamin D deficiency (for example, obesity, prolonged hospital admission or Black, Asian and Minority Ethnic groups) and adverse obstetric outcomes, and with randomization stratified by factors associated with the biochemical response to vitamin D supplementation^63–65^ and risk of poor labour outcomes.

Based on our findings, the number of women needed to treat with 1000 IU/day cholecalciferol to prevent one instrumental delivery is 14. As 1000 IU/day cholecalciferol for the duration of pregnancy for one woman in the UK costs ∼£15 (NHS prescribing cost of £1.45 for 30 tablets, British National Formulary 2021), the cost to prevent one instrumental delivery would be ∼£210. This, however, would be offset against the reduction in maternal and neonatal morbidity, and thus could be a relatively cheap intervention and warrants further investigation. If these findings and the other identified benefits of higher dose antenatal vitamin D supplementation such as increased offspring bone mass^66^ are replicated in further high quality randomized controlled trials without increased risk of harm, consideration should be given to increasing the recommended pregnancy supplementation guidance to 1000 IU/day in the UK. In the interim, promotion of the current guidelines recommending 400 IU/day vitamin D in pregnancy is appropriate to increase the current low uptake of supplementation.^67^

### Limitations of this study

A key limitation was the exclusion of women with 25(OH)D < 25 nmol/l in early pregnancy due to ethical and governance issues. Further intervention studies are, therefore, required in women with vitamin D deficiency who might particularly benefit from supplementation. Over 95% of the MAVIDOS participants were of White ethnicity, which reflects the local populations from which recruitment occurred but limits the generalisability of the study findings. Data on clothing choices, UVB exposure, dietary intake of vitamin D and previous pregnancy complications and outcomes were not collected. Nonetheless, considering the randomised controlled trial design of the study and inclusion of all women in the outcomes assessed in these analyses, random distribution of these characteristics between the two groups would be expected. These post hoc analyses are hypothesis-generating, rather than part of the pre-specified analysis plan for MAVIDOS, which primarily aimed to assess the effect of antenatal cholecalciferol supplementation on offspring bone development.^44^ However, as one of the largest trials of vitamin D supplementation in pregnancy, the MAVIDOS trial provides a unique opportunity to assess the effects of vitamin D supplementation in pregnancy on other outcomes, and preterm birth and delivery mode were chosen based on inconsistent findings in previously published observational studies that highlighted the need for data from intervention studies.

## Conclusions

In conclusion, in women with a baseline 25(OH)D 25-100 nmol/l, 1000 IU/day cholecalciferol during pregnancy did not reduce the incidence of preterm birth but was associated with a modest increase in the proportion who achieved a SVD and reduction in instrumental deliveries. Further trials are required to confirm this finding, and in particular, including women with very low levels of 25(OH)D at baseline.

## Data Availability

Data is not publicly available. Requests for data access will be considered on application to Prof N Harvey.

## Notes

**Clinical Trial Registry number:** ISRCTN:82927713; EUDRACT:2007-001716-23.

**Rebecca J. Moon,** NIHR Academic Clinical Lecturer in Child Health

**S. D’Angelo,** Statistician

**S.R. Crozier,** Statistician

**E.M. Curtis,** Associate Professor in Rheumatology

**M. Fernandes,** Academic Clinical Lecturer & MRC Clinical Research Training Fellow in Paediatrics

**A.J. Kermack,** NIHR Academic Clinical Lecturer in Obstetrics and Gynaecology

**J.H. Davies,** Consultant in Paediatric Endocrinology

**K.M Godfrey,** Professor of Epidemiology and Human Development

**N.J. Bishop,** Professor of Paediatric Metabolic Bone Disease

**S.H. Kennedy,** Professor of Reproductive Medicine and Director of the Oxford Maternal and Perinatal Health Institute

**A. Prentice,** Honorary Senior Visiting Fellow

**I. Schoenmakers,** Senior Lecturer

**R. Fraser,** Professor

**S.V. Gandhi,** Consultant in Obstetrics and Gynaecology

**H.M. Inskip,** Emeritus Professor of Statistical Epidemiology

**M.K. Javaid,** Associate Professor in Metabolic Bone Disease

**A.T. Papageorghiou,** Professor of Obstetrics and Fetal Medicine

**C. Cooper,** Professor of Rheumatology and Director of the MRC Lifecourse Epidemiology Unit

**N.C. Harvey,** Professor of Rheumatology and Clinical Epidemiology
